# Thoracic sympathetic block with botulinum toxin A for complex regional pain syndrome: a retrospective case series

**DOI:** 10.1016/j.inpm.2026.100774

**Published:** 2026-05-25

**Authors:** Jan van den Brink, Daniël P.C. van der Spek, Camiel J. Lalmohamed, Frank J.P.M. Huygen, Maaike Dirckx

**Affiliations:** Department of Anesthesiology, Center for Pain Medicine, Erasmus MC University Medical Center, Rotterdam, the Netherlands

**Keywords:** Complex regional pain syndrome, Sympathetic block, Botulinum toxin, Interventional pain management, Case series

## Abstract

**Background:**

Sympathetic interventions are frequently used in complex regional pain syndrome (CRPS). However, the evidence is inconclusive, and the response may depend on phenotype, anatomical target, and treatment durability. For upper-extremity CRPS, clinical data on thoracic sympathetic block (TSB) with botulinum toxin type A (BoNT-A) are lacking.

**Objective:**

To describe patient characteristics, procedural details, and clinical and thermographic outcomes after computed tomography (CT)-guided TSB with BoNT-A in upper-extremity CRPS.

**Methods:**

We performed a retrospective single-center case series of consecutive adults with upper-extremity CRPS who underwent CT-guided TSB with BoNT-A between January 2023 and March 2026. For each procedure, medical records were reviewed to descriptively assess pain change, duration of effect, repeat procedures, thermographic change, and adverse events.

**Results:**

Six patients underwent 7 procedures. Most suffered from persistent, treatment-refractory CRPS. Responses varied: 3 patients experienced a clinically meaningful analgesic benefit, 1 had thermographic improvement without clear analgesic benefit, and 2 had no meaningful response. No serious procedure-related complications were observed.

**Conclusion:**

CT-guided TSB with BoNT-A was feasible in this small retrospective series of refractory upper-extremity CRPS. Responses were heterogeneous, and thermographic improvement was not always accompanied by pain relief. The findings may prompt hypotheses regarding phenotype-based patient selection and the role of thermography.

## 1 Introduction

Sympathetic blocks are widely used in complex regional pain syndrome (CRPS), but their clinical value remains uncertain [1]. This uncertainty likely reflects variability in the underlying mechanisms of the syndrome [2]. CRPS presents as a disabling pain disorder that typically develops after trauma or surgery. It is characterized by continuing pain disproportionate to the inciting event. The pain is regional and accompanied by a variable combination of sensory, vasomotor, sudomotor/edema, and motor/trophic abnormalities [3,4]. Vasomotor disturbances such as color change and limb temperature asymmetry are prominent in a subset of patients. These features may reflect heterogeneous autonomic, adrenergic, inflammatory, and microvascular mechanisms [2,[5], [6], [7], [8]]. This makes vasomotor features relevant when considering sympathetic blockade, because this intervention specifically targets autonomic and adrenergic pathways. Their presence in only a subset of patients may therefore contribute to variability in treatment response and highlights the importance of considering clinical phenotypes in relation to sympathetic blockade [9].

Although this phenotype-directed approach for CRPS is clinically appealing, the evidence supporting sympathetic blockade remains limited. In a Cochrane review, the available randomized evidence for local anesthetic sympathetic blockade (LASB) was too sparse and too small to draw firm conclusions regarding efficacy [10]. Nevertheless, not all sympathetic interventions appear equally effective. In one notable randomized controlled trial (RCT), thoracic sympathetic block (TSB) as an adjuvant to standardized treatment was associated with less pain at 12 months compared to a control procedure, suggesting that targeted sympathetic intervention may still have a role in selected patients [11]. A major limitation of conventional LASB is its short-lived effect, prompting interest in more durable approaches. Botulinum toxin type A (BoNT-A) represents one such approach, as it may provide longer-lasting effects than local anesthetics. By inhibiting neurotransmitter release, BoNT-A may modulate nociceptive and autonomic signaling near the sympathetic ganglion [12]. In an RCT in lower-extremity CRPS, lumbar sympathetic block (LSB) with BoNT-A resulted in a greater increase in limb temperature and greater pain reduction over 3 months than local anesthetic alone [13]. For upper-extremity CRPS, targeting the thoracic sympathetic nerves may be particularly relevant, as the sympathetic innervation of the arm is anatomically variable. It includes accessory thoracic connections, such as the nerve of Kuntz, which may not be fully reached by a stellate ganglion block (SGB) [14]. Consistent with this rationale, a CRPS-specific randomized crossover trial demonstrated greater sympatholysis and better short-term clinical outcomes with T2 paravertebral sympathetic block than with SGB in upper-extremity CRPS [15]. Similarly, a multicenter randomized trial in chronic upper-extremity pain, including CRPS and neuropathic pain, found a higher rate of sympathetic blockade and greater immediate pain relief with ultrasound-guided T2 thoracic paravertebral block than with SGB [16].

To the best of our knowledge, there is no direct clinical evidence for TSB with BoNT-A in upper-extremity CRPS. Therefore, we present a retrospective case series of patients with upper-extremity CRPS treated with TSB and BoNT-A at our center, describing patient characteristics and changes in pain and limb temperature in daily clinical practice.

## 2 Methods

### 2.1 Study design and participants

This retrospective single-center case series included consecutive patients with upper-extremity CRPS who underwent computed tomography (CT)-guided TSB with BoNT-A at the Center for Pain Medicine of the Erasmus MC University Medical Center between January 2023 and March 2026. Patients were eligible if they were aged 18 years or older and had CRPS diagnosed according to the Valencia consensus-based adaptation of the International Association for the Study of Pain diagnostic criteria [3]. Procedures combined with other therapeutic injectates or co-interventions intended to modify analgesic outcome were excluded. Eligible cases were identified from a preexisting electronic medical record list of patients who had undergone the procedure at our center. All eligible patients who provided written informed consent were included.

### 2.2 Data collection

Eligibility was screened by JB in consultation with the treating pain physician. Data were extracted by JB and CL from the electronic patient record. Extracted variables included demographics, affected extremity/extremities, disease duration, CRPS characteristics, prior CRPS treatments, analgesic use, procedural details, adverse events and complications, repeat procedures, additional therapies, functional notes, pain outcomes, and thermographic images when available.

### 2.3 Thoracic sympathetic block procedure

Thoracic sympathetic blocks were performed under CT guidance according to our standard institutional protocol. CT guidance was used because the upper thoracic paravertebral target region is anatomically narrow and located close to the pleura. This allowed cross-sectional planning of the needle trajectory and direct visualization of the needle tip at the intended target. Patients were positioned prone on a positioning cushion, with the arms directed cranially. A planning CT scan was performed to identify the upper thoracic vertebral levels of the affected side. The final target level, either T2 or T3, was selected based on technical accessibility, including the anticipated soft-tissue trajectory and needle access. The skin entry point and craniocaudal needle trajectory were planned on CT images. Alignment was performed using the CT laser guidance system.

**Fig. 1:**
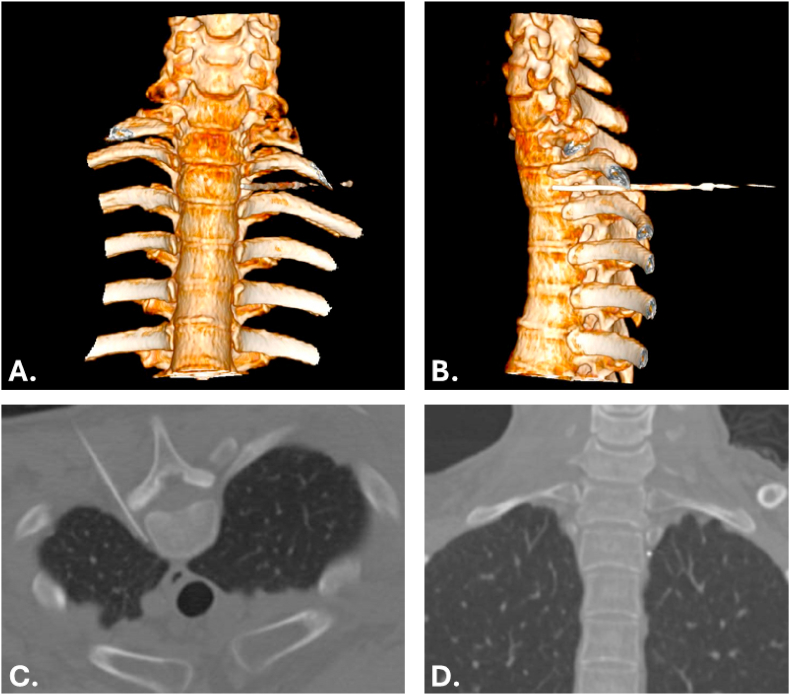
CT-guided TSB. A, Three-dimensional volume-rendered anterior view. B, Three-dimensional oblique/lateral view showing the needle trajectory. C, Axial CT image at the intervention level. D, Coronal visualization of the needle tip position.

After sterile skin preparation and draping, the intended skin entry site was infiltrated with local anesthetic. A 14-gauge intravenous cannula was inserted as an introducer sheath. Through this sheath, a 20-gauge, 145 mm blunt curved RF insulated cannula was advanced stepwise under intermittent CT guidance toward the thoracic sympathetic chain at the selected level (OWL Blunt Curved RF Insulated Cannula, REF 466-020/150/10-BC, DIROS Technology Inc., Markham, Ontario, Canada). The target was the anterior one-third of the vertebral body. No contrast was used. Correct placement was based on direct CT visualization of the needle tip at the planned target location. Fig. 1 shows 4 CT-based views of the needle trajectory and final needle tip position.

After confirmation of final needle position and negative aspiration, BoNT-A was injected. BoNT-A was administered as BOTOX® 100 Allergan units, powder for solution for injection (AbbVie B.V., Hoofddorp, the Netherlands). The product was reconstituted with 2 mL of sterile 0.9% sodium chloride. The intended dose was 100 Allergan units in 2 mL. However, when this volume could not be safely injected at the target level without backflow toward the intervertebral foramen, 50 Allergan units in 1 mL was administered. After the intervention, a postprocedural chest radiograph with inspiratory and expiratory views was obtained to evaluate for pneumothorax.

### 2.4 Thermography

Routine-care thermographic images were acquired using a FLIR T1020 camera. Acclimatization before image acquisition was part of standard clinical practice. Thermographic images were independently evaluated by JB and DS. Visual interpretation focused on side-to-side temperature differences between the affected and contralateral hand. Disagreements were resolved by consensus.

### 2.5 Outcome measures and reporting

Outcomes were assessed per procedure and included change in pain, duration of benefit, repeat procedures, thermographic change, and adverse events. Baseline was defined as the assessment recorded closest to the time of the procedure. Due to the retrospective design, follow-up was not restricted to predefined time points. All available postprocedural observations were collected from medical records. Pain was preferably reported using an 11-point numeric rating scale (NRS 0-10). When no numeric score was documented, qualitative descriptions of change were recorded.

Given the descriptive nature of this case series, no formal hypothesis testing was performed. Outcomes were reported case by case. Reasons for missingness were described when evident from medical records.

### 2.6 Ethics statement

This study was approved by the Medical Ethics Committee of the Erasmus MC (MEC-2025-0713). All included patients have provided written informed consent for the use of their clinical data and images.

## 3 Results

Between 2023 and 2026, 6 consecutive patients with upper-extremity CRPS underwent CT-guided TSB with BoNT-A. All patients met the eligibility criteria and provided written informed consent. Cases are presented in chronological order of treatment. Baseline characteristics, procedural details, and observed outcomes are summarized in Table 1.

Case 1 (analgesic response)

**Table 1:**
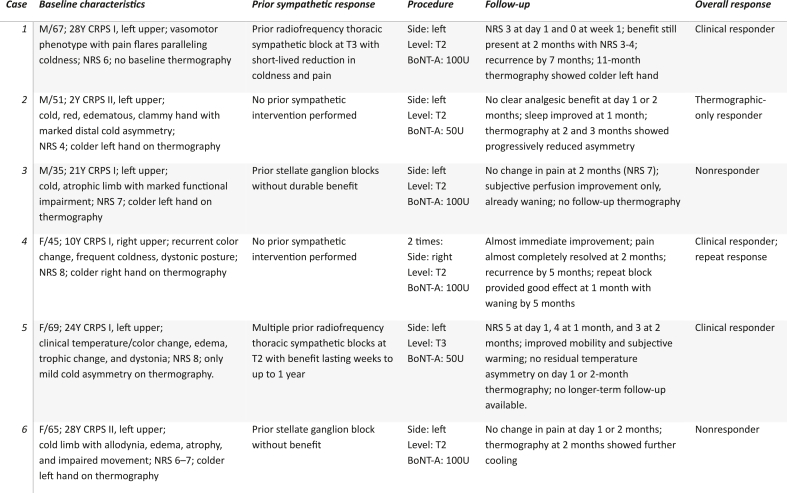
Baseline characteristics, prior sympathetic treatment history, procedural details, and outcomes after CT-guided thoracic sympathetic block with botulinum toxin type A for upper-extremity complex regional pain syndrome. Abbreviations: BoNT-A, botulinum toxin type A; CRPS, complex regional pain syndrome; F, female; M, male; NRS, numeric rating scale; T2/T3, thoracic target level.

A 67-year-old man had a 28-year history of CRPS type I of the left upper extremity. Prominent coldness of the affected limb was present, and pain flares closely paralleled these temperature changes. Baseline pain was NRS 6. Baseline thermography was unavailable. Previous treatments included intravenous mannitol, ketanserin, and ketamine; epidural electrical stimulation, and prior radiofrequency TSB at T3. The TSB had resulted in only a short-lived reduction in coldness and temporary pain relief. He underwent left-sided TSB at T2 with 100 U BoNT-A. Pain decreased to NRS 3 after day 1 and to NRS 0 after week 1, accompanied by subjective warming. Benefit was still present at 2 months, with pain reported as NRS 3-4. By 7 months, pain and coldness had returned. Thermography at 11 months showed a colder left hand, consistent with the recurrent clinical coldness.

Case 2 (thermographic improvement without analgesic response)

**Fig. 2:**
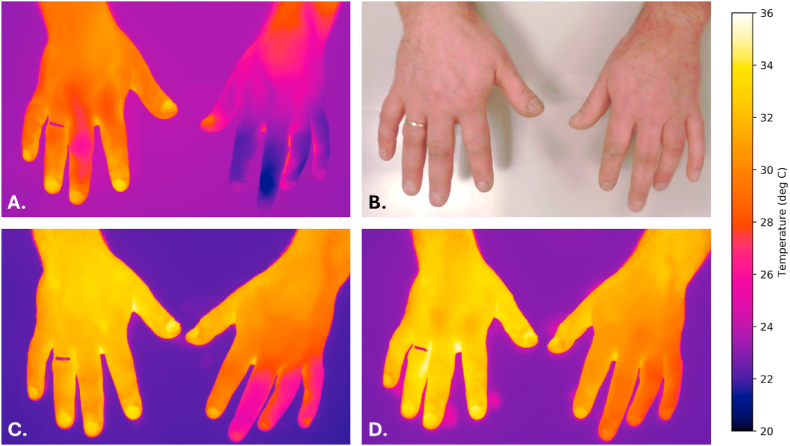
Serial thermographic and clinical images of both hands before and after intervention in Case 2. A, Thermographic image acquired before intervention. B, Clinical photograph of both hands before intervention. C, Thermographic image acquired 2 months after intervention. D, Thermographic image acquired 3 months after intervention.

A 51-year-old man had a 2-year history of CRPS type II of the left hand. Symptoms affected the left hand, wrist, and forearm, with sensory disturbance, red discoloration, coldness, edema, clamminess, and marked functional impairment. The hand felt clinically colder than the contralateral side, with thermography confirming marked distal cold asymmetry. Baseline pain was NRS 4. Before TSB, he had undergone multidisciplinary rehabilitation. He underwent left-sided TSB at T2 with 50 U BoNT-A. No pain relief was reported after day 1. There was no clear clinical benefit after 2 months, although sleep had improved after 1 month. In contrast, thermography after 2 months showed markedly reduced asymmetry, with further reduction after 3 months despite the absence of clear analgesic benefit. Serial thermographic and clinical images are shown in Fig. 2.

Case 3 (no analgesic response)

A 35-year-old man had a 21-year history of CRPS type I of the left upper extremity. The limb felt cold and was severely affected by atrophy, marked functional impairment, allodynia, and hyperalgesia. Thermography confirmed the cold asymmetry. Baseline pain was NRS 7. Previous treatments included multidisciplinary rehabilitation, (co-)analgesics, stellate ganglion blocks, intravenous ketamine, and spinal cord stimulation, without durable benefit. He underwent left-sided TSB at the T2 level with 100 U BoNT-A. At 2 months, pain remained unchanged at NRS 7. He reported a slight, subjective warming of the affected arm, but this effect was already diminishing at the follow-up. No follow-up thermography was available.

Case 4 (analgesic response with repeated benefit)

A 45-year-old woman had a 10-year history of CRPS type I of the right upper extremity. Symptoms included recurrent skin color change, frequent coldness, and dystonic posturing. Thermography showed cold asymmetry. Baseline pain was NRS 8. Previous treatments included rehabilitation-based interventions, (co-)analgesics, intravenous therapies with mannitol and pamidronate, and epidural electrical stimulation. These were initially effective but later lost efficacy. She underwent right-sided TSB at T2 with 100 U BoNT-A. Improvement occurred almost immediately. After 2 months, pain had almost completely resolved. Symptoms recurred after 5 months. Seven months after the initial procedure, TSB with BoNT-A was repeated at the same level and dose, again with good effect at 1-month follow-up. Five months after the repeat procedure, the patient reported that the effect was diminishing. The pain increased to NRS 6 and the limb became more swollen, darker in color, and colder. No follow-up thermography was available.

Case 5 (analgesic response)

A 69-year-old woman had a 24-year history of CRPS type I of the left upper extremity. Symptoms included allodynia, hyperalgesia, clinical temperature asymmetry, intermittent red and blue discoloration, edema, trophic change, and dystonia. Baseline pain was NRS 8. Thermography showed only mild cold asymmetry. Previous treatments included (co-)analgesics, infusion therapies, and multiple radiofrequency TSBs at T2. The TSBs had provided pain relief lasting from several weeks to up to 1 year. She underwent left-sided TSB at T3 with 50 U BoNT-A. After 1 day, pain had decreased to NRS 5, with improved mobility and subjective warming of the hand. Thermography performed at the same time showed no residual temperature asymmetry. After 1 month, pain remained improved at NRS 4. At 2 months, pain had further improved to NRS 3 and tramadol use had substantially decreased. Thermography was symmetrical. Longer-term follow-up was not available.

Case 6 (no analgesic response)

A 65-year-old woman had a 28-year history of CRPS type II of the left upper extremity. Symptoms included allodynia, hyperalgesia, edema, atrophy, and impaired movement. The affected limb was clinically and thermographically colder than the contralateral side. Baseline pain was NRS 6-7. Previous treatments included rehabilitation, (co-)analgesics, pamidronate and ketamine infusions, and stellate ganglion block. None provided meaningful or durable benefit. She underwent left-sided TSB at T2 with 100 U BoNT-A. Pain was unchanged at the early postprocedural assessment and remained unchanged from baseline after 2 months, with NRS 6-7. Follow-up thermography showed further cooling of the affected side.

### 3.1 Complications and adverse events

No serious procedure-related complications were observed in the 7 procedures. Minor or transient events included postprocedural pain. In Case 1, one episode of light-headedness, dizziness, nausea, blurred vision, and headache occurred after the procedure. This was investigated and considered unrelated to the intervention. In Case 2, transient bluish discoloration of the third digit was observed.

## 4 Discussion

To the best of our knowledge, this is the first report of TSB with BoNT-A in upper-extremity CRPS. In our retrospective series, CT-guided TSB with BoNT-A was performed in 6 tertiary referral patients with predominantly long-standing, treatment-refractory CRPS. Within this highly selected population, responses were heterogeneous. Three patients showed clinically meaningful improvement, one showed objective thermographic improvement without clear analgesia, and two did not improve. In the responders, benefit emerged rapidly, within 24 h to 1 week and lasted from several weeks to months. No serious procedure-related complications occurred.

These observations should be considered in the context of heterogeneous evidence for sympathetic blockade in CRPS [10,13]. The limited evidence for effectiveness of conventional LASB may partly reflect heterogeneity in patient selection, anatomical target, and duration of blockade, rather than the absence of therapeutic effect. In upper-extremity CRPS, thoracic sympathetic targeting may provide more reliable sympatholysis than SGB [15,16]. Separately, the short duration of LASB provides a rationale for using longer-acting adjuncts such as BoNT-A. BoNT-A has shown prolonged effects in studies of LSB for lower-extremity CRPS [17,18]. The present series brings these concepts together by applying BoNT-A to CT-guided TSB in upper-extremity CRPS. Given the small, retrospective, and highly selected nature of this series, no firm conclusions regarding effectiveness can be drawn. However, the observed heterogeneity in response may give rise to hypotheses regarding the involvement of the sympathetic nervous system and the potential role of thermographic assessment in patient selection and response evaluation.

The heterogeneous response pattern may support a phenotype-directed rather than disease-directed interpretation. Sympathetic involvement in CRPS is complex and may vary with disease duration, vasomotor phenotype, and peripheral tissue changes. Early warm or vasodilatory presentations may reflect impaired cutaneous sympathetic vasoconstrictor activity rather than sympathetic overactivity [8,19]. Later cold presentations may involve secondary neurovascular changes [5,8]. At the peripheral tissue level, altered adrenergic responsiveness has also been demonstrated in CRPS subgroups, including upregulation of α1-adrenoceptors on epidermal cells and cutaneous nerve fibers [6,8,[19], [20], [21]]. This peripheral adrenergic sensitization may allow sympathetic signaling to remain coupled to pain, even without increased sympathetic outflow [7,21]. Inflammatory pathways may further amplify this coupling, as experimental work links α1-adrenoceptor signaling to IL-6 mediated inflammatory responses [22,23]. In this context, sympathetic intervention may be most relevant when an adrenergic, inflammatory, and neurovascular pain state is still modifiable, rather than when fixed trophic, motor, or central changes predominate. This raises the possibility that earlier intervention may be associated with a greater likelihood of clinical benefit. Consistent with this interpretation, responses in our series did not appear to follow temperature asymmetry alone. Less favorable outcomes tended to occur in patients with marked atrophy, severe loss of function, or impaired movement, despite thermographic asymmetry. In contrast, beneficial outcomes were observed in patients whose vasomotor features remained dynamic or who had previously exhibited a sympathetic response.

A second important observation is the imperfect coupling between temperature change and pain relief. One patient showed marked thermographic improvement without clear analgesia, whereas another reported subjective warming without meaningful pain reduction. This dissociation is consistent with prior studies showing that temperature change after sympathetic intervention correlates weakly with immediate pain relief and does not reliably predict sustained outcome [24,25]. Importantly, a postprocedural temperature increase should not be interpreted as proof of complete upper-extremity sympatholysis. Partial sympatholysis may still produce measurable warming. Because contrast was not used, craniocaudal spread of the injectate along the upper thoracic sympathetic chain could not be verified. Therefore, complete coverage of sympathetic outflow to the upper extremity could not be confirmed. Thermography should therefore be viewed as an imperfect physiologic marker of regional perfusion and possible sympathetic target engagement, not as a surrogate endpoint for clinical success. Endothelial dysfunction may partly explain this discrepancy. Cold CRPS phenotypes have been associated with increased endothelin-1, reduced nitric oxide metabolites, and impaired endothelium-dependent vasodilation [5,26]. It is therefore possible that the temperature improves while pain persists if endothelial, inflammatory, motor, or central mechanisms predominate. Conversely, persistent or worsening cold asymmetry after TSB could reflect incomplete sympatholysis or block failure. In long-standing CRPS, this finding may also reflect fixed neurovascular or trophic changes.

The retrospective, uncontrolled, and small nature of this series limits its interpretation to hypothesis generation. The cohort was drawn from a tertiary referral practice and consisted mainly of heavily pretreated patients with persistent CRPS. This increases relevance for refractory cases but limits generalizability to the broader CRPS population. Missing data, particularly for serial NRS scores and repeat thermography, further limit interpretation. Many patients traveled long distances to our center, resulting in telephone-based follow-up. This reduced outcome granularity and limited repeat imaging. Accordingly, these data should be interpreted as an exploratory indication regarding feasibility, phenotype, and potential responder characteristics, not as an estimate of effectiveness.

## 5 Conclusions

In this case series, CT-guided TSB with BoNT-A was feasible in highly selected patients with refractory upper-extremity CRPS and was not associated with serious procedure-related complications. Analgesic benefit was observed in a subset of patients but response was inconsistent and thermographic changes did not reliably mirror pain outcomes. From a practical perspective, the findings support further study of phenotype-based selection, prior sympathetic response, and the role of thermography in distinguishing target engagement from clinical benefit.

## Patient consent statement

Informed consent was obtained from all patients.

## Author contributions

JvdB is the guarantor of the article, involved in conceptualization, collection of data, data analysis, writing of the manuscript and approval of the definitive version. DvdS was involved in conceptualization, collection of data, data analysis, writing of the manuscript and approval of the definitive version. CL was involved in collection of data, data analysis, critical review of the manuscript and approval of the definitive version. FH was involved in conceptualization, data interpretation, critical review of the manuscript and approval of the definitive version. MD was involved in conceptualization, collection of data, data interpretation, critical review of the manuscript and approval of the definitive version.

## Data availability statement

The data that support the findings of this study are not publicly available because they contain information that could compromise the privacy of the research participants. De-identified data may be made available from the corresponding author upon reasonable request, subject to applicable institutional requirements and Dutch privacy regulations.

## Ethics approval statement

This study was reviewed under the Dutch non-WMO framework and was determined not to fall within the scope of the Medical Research Involving Human Subjects Act (WMO) by Niet WMO Toetsingscommissie Erasmus MC under reference number MEC-2025-0713. All participants provided written informed consent prior to participation.

## Declaration of generative AI and AI-assisted technologies in the manuscript preparation process

During the preparation of this manuscript, the authors used ChatGPT (OpenAI) to assist with improving language and readability. The authors reviewed and edited the output as needed and take full responsibility for the content of the published article.

## Funding

This research did not receive any specific grant from funding agencies in the public, commercial, or not-for-profit sectors.

## Conflicts of interest

The authors declare that they have no conflict of interest regarding the publication of this study.
